# Transcriptomic, cytological, and physiological analyses reveal the potential regulatory mechanism in Tartary buckwheat under cadmium stress

**DOI:** 10.3389/fpls.2022.1004802

**Published:** 2022-10-12

**Authors:** Xueling Ye, Qiang Li, Changying Liu, Qi Wu, Yan Wan, Xiaoyong Wu, Gang Zhao, Liang Zou, Dabing Xiang

**Affiliations:** Key Laboratory of Coarse Cereal Processing, Ministry of Agriculture and Rural Affairs, Sichuan Engineering and Technology Research Center of Coarse Cereal Industrialization, School of Food and Biological Engineering, Chengdu University, Chengdu, China

**Keywords:** Tartary buckwheat, Cd stress, regulatory pathway, RNA-Seq, root transcriptome

## Abstract

Rapid industrialization and urbanization have caused serious cadmium (Cd) pollution in soil. Tartary buckwheat is an important pseudocereal crop with the potential ability to tolerate various stresses. However, the responses to Cd stress in this species are unclear. In this study, we assessed the phenotypic, cytological, physiological, and transcriptomic characteristics of Tartary buckwheat under the various concentrations of Cd treatments to investigate the responses and their regulatory pathways for the first time. The results showed Tartary buckwheat could tolerate the high Cd concentration of 50 mg/L under Cd stress. The average root diameters increased as a result of more cell layers of the endodermis and the bigger size of the pericycle. Cd primarily accumulated in roots and relatively less transferred to leaves. Antioxidant activities and malondialdehyde (MDA) accumulation varied in different tissues and different Cd concentrations of treatments. Meanwhile, Cd stress led to the formation of Casparian strips in roots and damaged the cytoderm and organelles. The weighted gene co-expression and interaction network analyses revealed that 9 core genes induced by Cd stress were involved in metal ion binding, Ca signal transduction, cell wall organization, antioxidant activities, carbohydrate metabolic process, DNA catabolic process, and plant senescence, which regulated a series of phenotypic, cytological, and physiological changes above. These results laid the foundation for a deep understanding of the responses to Cd toxicity in Tartary buckwheat. It’s also a critical reference for the functional characterization of genes for Cd tolerance.

## Introduction

With the rapid development of industry and agriculture, the ecological environment has deteriorated. Heavy metal pollution in the environment is a serious threat to plants and animals. It also causes human health problems with the food chain. Of them, cadmium (Cd) has been the main pollute element as its toxicity, bio-persistence, superior absorption by plants, and widespread exists in the environment (Qin et al., 2020). Many countries like China, India, Japan, America, Germany, and Belgium have suffered from Cd pollution (Kubier et al., 2019). According to the reports on the State of the Ecology and Environment in China (2019) and National Soil Pollution Survey Bulletin (2014), Cd has become the main pollutant in heavy metal pollution since 2019 and around 7.0% of farmland has exceeded the allowable Cd contamination level (Zhao et al., 2015; MEE, 2020). Cd is widely found in a range of sources like batteries, electroplating, plastics, coating, pesticides, fertilizers, and wastewater that are closely related to people’s lives (Bali et al., 2020). As a stable pollutant, Cd could be accumulated and retained in the soil for decades. Meanwhile, Cd is easily absorbed and transported in plants due to its good activity in the soil (Shanmugaraj et al., 2019). Excess absorption of Cd could cause serious toxicity to plants at the morphological, cytological, physiological, biochemical, and molecular levels (El Rasafi et al., 2020). Cd in the soil can directly contact plant roots, resulting in the inhibition of root growth and the absorption of water and nutrients (Pál et al., 2006; Abedi and Mojiri, 2020). Cd pollution also causes leaf yellowing and senescence, and biomass reduction (Rizwan et al., 2016; El Rasafi et al., 2020). Cd toxicity further results in metabolic dysfunction, inhibition of chlorophyll, organic matter, and protein synthesis, reduction of photosynthesis and respiration, and changes in enzyme activity, thus causing a decline in crop yield ad quality (Pál et al., 2006; Rizwan et al., 2016; Abedi and Mojiri, 2020; El Rasafi et al., 2020), which finally leads to serious food security problems.

The toxic effects of Cd have been widely reported in many crops like rice, wheat, maize, potatoes, oilseed rape, and soybean (Pál et al., 2006; Rizwan et al., 2016; Qin et al., 2018). Physiological and molecular mechanism of Cd stress in these crops has been intensively studied (Pál et al., 2006; Rizwan et al., 2016; Abedi and Mojiri, 2020; Fan et al., 2021). Many antioxidant enzymes like peroxidase (POD), superoxide dismutase (SOD), ascorbate peroxidase (APX), glutathione peroxidase (GPX), catalase (CAT), and nonenzymatic antioxidants glutathione (GSH) play important roles in reducing reactive oxygen species (ROS) that may help alleviate oxidative stress. The malondialdehyde (MDA) is an important indicator in assessing the degree of lipid peroxidation under oxidative damage (Rizwan et al., 2016; Rizwan et al., 2017; Ahmad et al., 2019; Chen et al., 2020; El Rasafi et al., 2020). To deal with Cd stress, plants have developed various strategies classified into three categories: avoidance through limiting the Cd uptake, tolerance by accumulation and sequestration of Cd in some special parts like vacuole, and resistance of Cd toxicity by the antioxidant system (El Rasafi et al., 2020). These strategies depend on species, genotypes, environments, and many other factors (Rizwan et al., 2017; El Rasafi et al., 2020). Breeding Cd tolerance genotype is an efficient, safe, and environmental strategy to maintain the quality and yield under its stresses. Studying the responses to Cd stress and understanding their regulatory pathways provide a theoretical reference for screening and characterizing Cd resistance plants.

Tartary buckwheat (*Fagopyrum tataricum* Gaertn.) is a dicotyledonous herb belonging to the Polygonaceae family and *Fagopyrum* genus, an important pseudocereal crop with properties both of food and medicine (Zou et al., 2021). Tartary buckwheat is a short-season crop, widely grown up at high altitudes, in cool and humid environments, which has the characteristics of rapid growth, strong adaptability, and high tolerance to harsh and barren environments. It’s also famous for its high content of flavonoids. D’Amelia et al. (2018) reported flavonoid is a kind of antioxidant that can remove ROS resulting from the biotic and abiotic stress to reduce damage in plants. Wang et al. (2015) found that Tartary buckwheat can detoxify aluminum (Al) by forming non-phytotoxic Al complexes with organic acids. Meanwhile, Tartary buckwheat can improve the tolerance to lead stress by sequestrating lead in leaf vacuoles (Wang et al., 2020). Yang et al. (2015) and Shi et al. (2019) identified that low and high concentrations of Cd treatments could promote and inhibit Tartary buckwheat seed germination, respectively. Lu et al. (2019) reported that endogenous sulfur can inhibit Cd accumulation in the leaves of Tartary buckwheat, but significantly increase Cd concentration in roots. All studies have focused on the phenotypic and physiological impacts caused by Cd stress, but the response to Cd stress on Tartary buckwheat at molecular levels is unclear now. China is the origin of Tartary buckwheat, that’s also the major producer and exporter in the world (Zhou et al., 2018). The annual cultivation area of buckwheat maintains more than 530,000 ha. Cd contamination occurs in many buckwheat growing areas in recent years (Huang et al., 2019). Thus, we should speed up the comprehensive and in-depth research of Tartary buckwheat from Cd stresses.

Transcriptome analysis is a powerful tool for the in-depth study of plants at the gene expression and molecular level. It has been used in the research of Tartary buckwheat under various abiotic stresses and revealed the regulation mechanisms. Wang et al. (2020) identified the fundamental lead tolerance, and explored hyperaccumulation mechanisms regulated by TFs, metal iron binding, and membrane transport proteins in the leaves of Tartary buckwheat through transcriptome profiling. Jeon et al. (2018) and Song et al. (2022) took advantage of transcriptomic analysis to demonstrate the phenylalanine pathway played a vital role in responding to cold stress. Huang et al. (2021) determined an ABA-dependent and ABA-independent pathway in the regulation of drought stress in Tartary buckwheat based on the transcriptome analysis. Also using transcriptome analysis, Song et al. (2021) found that ROS induced by salt stress acted as a signal messenger triggering ROS-regulated protein kinases and then caused the phosphorylation/dephosphorylation of transcription factors. And the increase of ROS content can promote the activities of SOD, POD, APX, and other antioxidant enzymes to enhance salt resistance. The application of transcriptome analysis in the study of Tartary buckwheat stress has been very mature. But it’s a blank in the research under Cd stress in Tartary buckwheat.

In this study, we investigated the phenotypes, primary-and ultrastructure of roots, antioxidant activities, the Cd concentration in different tissues, and the gene expression in Tartary buckwheat seedlings roots under various concentrations of Cd stress for the first time. The objectives of this study were to (1) understand the effects of Cd stress on the phenotypic, physiological, cytological, and transcriptomic characteristics in Tartary buckwheat seedlings, and (2) identify the regulatory pathway and the core genes in Tartary buckwheat under Cd stress. The comprehensive analysis of Tartary buckwheat under Cd stress provides the reference for understanding the mechanism of Cd response and the functional characterization of genes for Cd tolerance.

## Materials and methods

### Plant materials and experimental design

Tartary buckwheat used in this study is Xiqiao No. 1 (XQ1) derived from radiation-induced mutagenesis breeding. It’s a commercial variety characterized by early maturity and high yielding potential, widely planted in southwest China. The seeds were provided by the Key Laboratory of Coarse Cereals Processing of the Ministry of Agriculture and Rural Affairs at Chengdu University. The seeds of 40 g were disinfected in 1% potassium permanganate for half an hour and then rinsed with clean water. The cleaned seeds germinated on the wet gauze in sprouting trays in an illumination incubator for 4 days. The temperature, relative humidity, light intensity, and light cycle were 24/18°C (day/night), 70%, 150 μmol/m^2^/s, and 10/14 hours (day/night), respectively. Seedlings with similar coleoptile lengths were then selected and divided into four groups in sprouting trays containing filter paper with suitable holes. The sprouting trays were put in the different hydroponic systems with Hoagland solution (Hoagland and Arnon, 1950) containing 0, 10, 30, 50 mg/L CdCl_2_ (designated as CK, Cd10, Cd30, and Cd50), respectively. Three biological replicates were used for each treatment.

### Analysis of root morphological characteristics

After a 7-day treatment of Cd stress, the root morphological characteristics under different treatments were scanned using Epson XL (12000×) and evaluated using a WinRHIZO Pro 2017 image analysis software (Régent Instruments, Quebec, Canada). The root morphological characteristics included total root length (TRL), total root surface area (TRSA), average root diameter (ARD), total root volume (TRV), number of root tips (RT), and number of root forks (RF). Ten plants from each biological duplication were measured. The significant differences (at the level of *p* < 0.05) in root morphological characteristics among different treatments were analyzed using the software SPSS 20.0 (IBM Corp., Armonk, NY, USA) with the Duncan *t*-test.

### The transverse sections and ultrastructural examination of roots

To investigate the changes in the primary structure of roots under Cd stresses, we got the transverse sections of the root maturation zone through pretreatment, dehydration, embedding, and cutting. They were double-stained with Safranin O and Fast Green, examined using a light microscope (Nikon Eclipse E100, Tokyo, Japan) and imaging software (Nikon DS-U3, Tokyo, Japan). To examine their cell ultrastructure, the roots were fixed for 12 hours at 4°C in 0.1 M phosphate buffer (PB) containing 2.5% glutaraldehyde and then washed in PB. The roots were then fixed in PB containing 1% OsO_4_ for 1 hour, dehydrated in a graded series of ethanol, embedded in LR white resin, and then got the ultrathin sections (80-nm thick). The sections were examined using a transmission electron microscopic (TEM, Hitachi HT7700 TEM, Japan).

### Measurement of antioxidant activity and MDA

Antioxidants were closely related to biological and abiotic stress. Enzymatic antioxidants like SOD, POD and nonenzymatic antioxidants GSH play an important role in reducing ROS that may help alleviate oxidative stress. MDA is an important indicator for the degree of lipid peroxidation under oxidative damage. Together with MDA content, we assessed activities of SOD, POD, and GSH in different tissues (root, stem, and leaves) after 7-day treatment. All of these assessments were carried out using the corresponding assay kits (Jiancheng Biotechnology, Nanjing, China) with three biological duplications. The significant differences (at the level of *p* < 0.05) in values were analyzed using software SPSS 20.0 (IBM Corp., Armonk, NY, USA) with the Duncan *t*-test.

### Determination of Cd concentration in different tissues

The Cd concentration in different tissues was measured after 7-day treatment. Roots, stems, and leaves were collected with three biological replicates. The collected materials were dried in an electric thermostatic drying oven at 105°C for half an hour and then at 65°C for 48 hours. The dried materials were ground into powders with a diameter of less than 0.250 mm. 0.2 g of each sample was digested using a Graphite Block Acid Digestion System (ODLAB Co., Ltd., Seoul, Korea), then suspended in 50 ml of ultrapure water, filtered, and finally determined the Cd content using inductively coupled plasma-mass spectroscopy (ICP-MS) (iCAP RQ, Thermo Fisher Scientific Inc., Massachusetts, USA).

### RNA extraction, cDNA library preparation, and sequencing

Following 7-day treatments, the root tissues were sampled with three independent biological replicates and quickly frozen into liquid nitrogen. Total RNA was extracted using a Plant RNA Extraction kit (Takara Bio, Otsu, Japan) following the manufacturer’s protocol. RNA concentration and purity were measured using NanoDrop 2000 (Thermo Fisher Scientific, Wilmington, DE). RNA integrity was assessed using the RNA Nano 6000 Assay Kit of the Agilent Bioanalyzer 2100 system (Agilent Technologies, CA, USA). The RNA with the criterion of RIN larger than 8.5 were subjected to the subsequent construction of complementary DNA (cDNA) libraries. mRNAs were enriched from total RNA using oligo(dT)-attached magnetic beads, and then were randomly fragmented using divalent cations under elevated temperature in NEBNext First Strand Synthesis Reaction Buffer (5X). First strand cDNA was synthesized using random hexamer primer and M-MuLV Reverse Transcriptase. Second strand cDNA synthesis was subsequently performed using DNA Polymerase I and RNase H. Double-strand cDNA was subjected to end repair. Adenosine was added to the end and ligated to adapters. AMPure XP beads were applied here to select fragments within the size range of 300-400 bp. The cDNA library was obtained by certain rounds of PCR on the selected fragments. Paired-end 2×150 bp reads were generated using an Illumina platform (NovaSeq 6000).

### Transcriptome analysis

Based on the initial assessments using the software FastQC v 0.11.5 (Andrews, 2016), we removed the adapters from the obtained sequence reads, and deleted ploy-N (>10%) and low-quality reads (the bases number of Q ≤ 10 greater than 50%) from raw sequences using SolexaQA++ (Cox et al., 2010) to generate clean sequences with high quality. All the downstream analyses were based on the clean sequences with high quality. The clean sequences were mapped onto the reference genome of Tartary buckwheat Puku1 (http://www.mbkbase.org/Pinku1/) using HISAT2 v2.0.4 (Kim et al., 2015) with default parameters. The mapped reads were assembled using the StringTie v1.3.4d (Pertea et al., 2015). The gene expression values FPKM (fragments per kilobase of transcript length per million mapped reads) (Florea et al., 2013) were also calculated using StringTie v1.3.4d (Pertea et al., 2015). The criteria of RPKM≥1 were used to define expressed genes. The differentially expressed genes (DEGs) were identified using DESeq2 v1.6.3 in the R package (Love et al., 2014) based on a model using the negative binomial distribution with the criteria of Fold Change (FC) ≥ 2 and FDR (false discovery rate) < 0.01. Based on the reference genome sequences, the software StringTie v1.3.4d (Pertea et al., 2015) was used to assemble mapped reads and compared them with the original genome annotation to find the originally unannotated transcription regions. The transcript regions without annotation obtained by the above processes are defined as novel transcripts or new genes.

### Functional annotation, pathway enrichment analysis, and WGCNA analysis

To annotate the genes we identified, the software blastx 2.2.31 (Altschul et al., 1997) was used to search against the multiple databases including Swiss-Prot (A manually annotated, non-redundant protein sequence database) (Apweiler et al., 2004), GO (Gene Ontology) (Ashburner et al., 2000), KOG (Clusters of Protein homology) (Koonin et al., 2004), Pfam (Protein family) (Finn et al., 2014), and KEGG (Kyoto Encyclopedia of Genes and Genomes) (Kanehisa et al., 2004). The KEGG Orthology results were obtained using the software KOBAS2.0 (Xie et al., 2011). The predicted amino acid sequences of target genes were blasted against the Pfam database using software hmmscan 3.1b2 (Eddy, 1998) to gain the annotation information. The software KOBAS (Mao et al., 2005) was used to test the statistical enrichment of DEGs in KEGG pathways. The interaction networks among genes were visualized using the software Cytoscape 3.8.2 (Shannon et al., 2003). The software MapMan 3.6.0 (https://mapman.gabipd.org/mapman-version-3.6.0) was used to analyze and visualize the metabolic pathways. To study the Cd-induced genes and the related traits they regulate, we analyzed the relationship between specific traits (ARD and Cd concentration in different tissues) and key modules using the weighted gene co-expression network analysis (WGCNA) using WGCNA (v 1.0) package in R software (Langfelder and Horvath, 2008). All the 3,647 DEGs between CK and Cd stress were applied to WGCNA. The cutreeStatic function was used to remove the offending sample. The parameter minModuleSize was set to 30. Network visualization for each module was performed using the software Cytoscape 3.8.2 (Shannon et al., 2003).

### Quantitative real-time polymerase chain reaction (qRT-PCR)

The qRT-PCR was used to verify the expression level of Cd-induced genes in the roots of Tartary buckwheat seedlings. The reference gene was *FtH3* (Li et al., 2019). All primers were designed using the online system (https://www.sangon.com/newPrimerDesign). The cDNA was synthesized using the EasyScript One-Step gDNA Removal and cDNA Synthesis SuperMix kit (TransGen Biotech, Beijing, China) according to the manufacturer’s protocol. The qRT-PCR reactions were performed in qTOWER3 G Real-Time PCR System (Analytik Jena AG, Germany) following the procedure of 95°C for 30 s, followed by 40 cycles of 95°C for 10 s, 60°C for 30 s, and 72°C for 30 s. Finally, we used the 2^−ΔΔCt^ method to normalize the gene expression (Livak and Schmittgen, 2001).

## Results

### Morphological characteristics of roots

**Figure 1:**
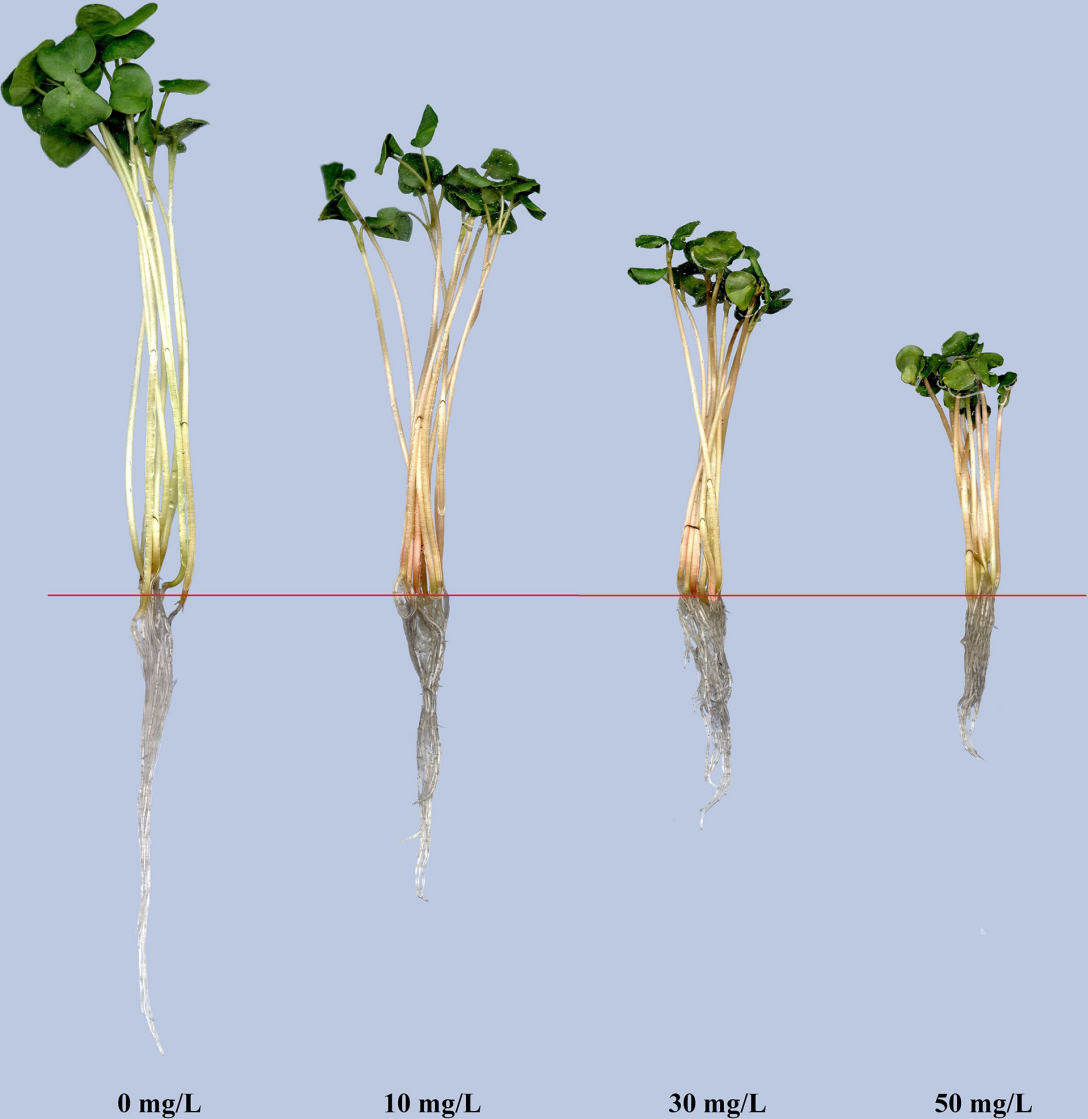
The Tartary buckwheat seedling under cadmium stress of various concentrations for 7-day treatments.

**Figure 2:**
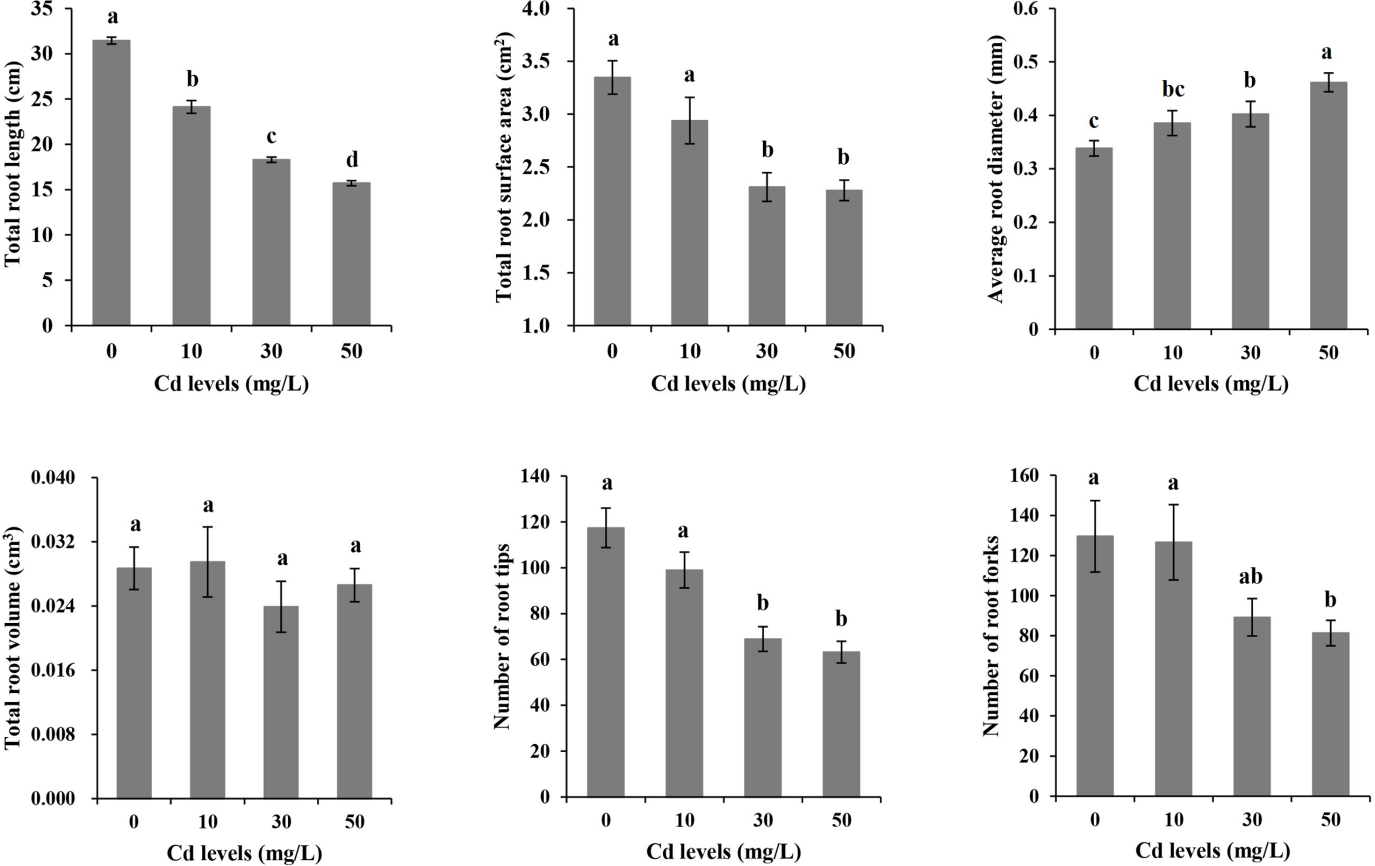
The morphological characteristics of roots in Tartary buckwheat seedling under cadmium stresses. Different lowercase letters on the top of bars indicate significant differences between treatments according to Duncan *t*-test (*p*-value < 0.05).

With the increase of Cd treatment concentration, the growth inhibition strengthened. Chlorosis and necrosis were not observed on plants although the stems turned red under Cd stress (**Figure 1**). We scanned the root morphological characteristics and analyzed TRL, TRSA, ARD, TRV, RT, and RF under each of the four different treatments (**Figure 2**). The average value for TRL was 31.456 cm, for TRSA was 3.3473 cm^2^, for ARD was 0.3384 mm, for TRV was 0.0287 cm^3^, for RT was 117.4, and for RF was 129.6 under CK treatment. Cd stress had no significant effects on TRV. TRL became significantly lower with the increase of the Cd concentration. No significant differences existed in TRSA, ARD, RT, or RF between CK and Cd10. So did between Cd30 and Cd50 except for TRL and ARD. There were no significant differences in ARD and RF between Cd10 and Cd30. TRSA and RT under Cd30 and Cd50 were significantly lower than that under CK and Cd10. RF was significantly lower under Cd50 than other treatments. To our surprise, ARD significantly increased under Cd30 and Cd50 compared with those in CK.

### Antioxidant activity and MDA accumulation

**Figure 3:**
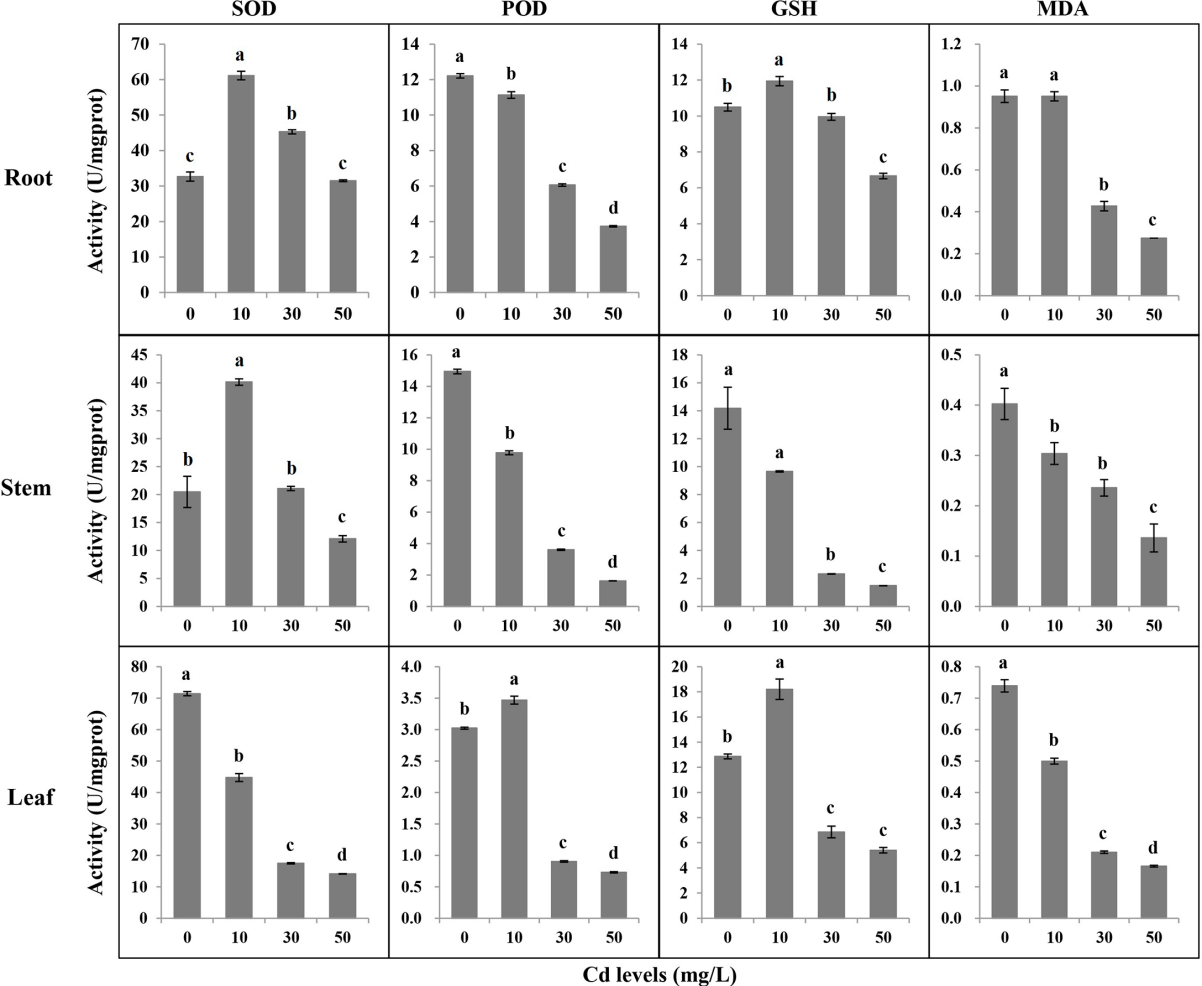
The antioxidant activities and malondialdehyde content in different tissues of Tartary buckwheat seedling after 7-day treatments. SOD, superoxide dismutase; POD, peroxidase; GSH, glutathione; MDA, malondialdehyde. Different lowercase letters on the top of bars indicate significant differences between treatments according to Duncan *t*-test (*p*-value < 0.05).

Cd can trigger the antioxidant system to respond to Cd toxicity in plants. We tested SOD, POD, GSH, and MDA in different tissues after a 7-day treatment of Cd stress. The antioxidant activities and MDA accumulation varied in different tissues under different Cd stresses (**Figure 3**). In roots, the activities of SOD under Cd10 and Cd30, and the GSH under Cd10 increased, while the GSH decreased under Cd50. The activities of POD and the accumulation of MDA significantly decreased with the increase of Cd concentration. In stems, the higher the Cd concentration, the lower the activities of POD and GSH, and the less the accumulation of MDA. The activities of SOD significantly increased and decreased under Cd10 and Cd50, respectively. In leaves, the activities of SOD and the accumulation of MDA significantly decreased with the increase of Cd concentration. The activities of POD and GSH significantly increased under Cd10 but significantly decreased under Cd30 and Cd50.

### Cd concentration in different tissues

**Figure 4:**
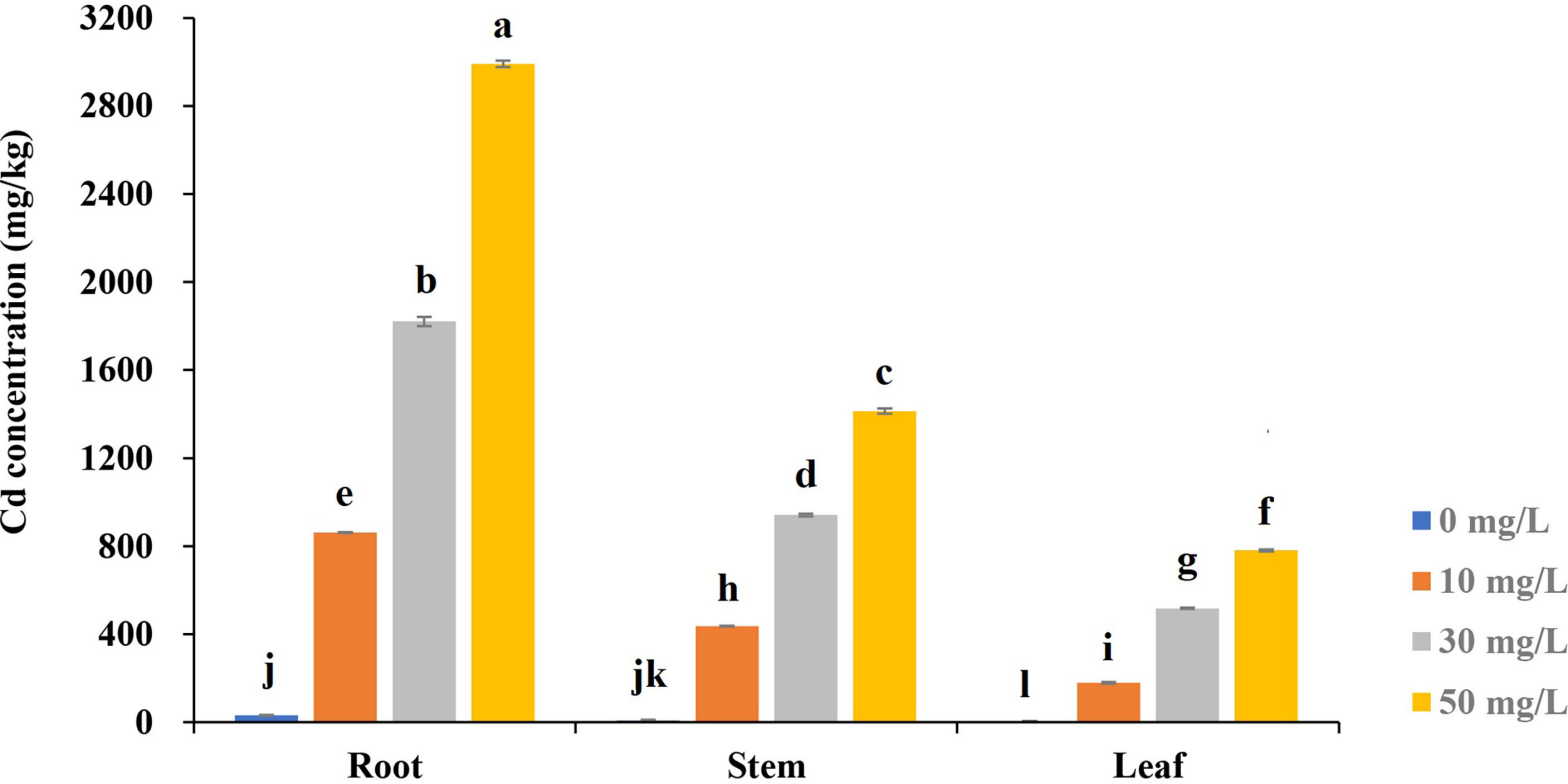
The Cd concentration in different tissues of Tartary buckwheat seedling under various cadmium stresses after 7-day treatments. Different lowercase letters on the top of bars indicate significant differences between treatments according to Duncan *t*-test (*p*-value < 0.05).

We analyzed the Cd accumulation in roots, stems, and leaves under different concentrations of Cd treatment (**Figure 4**). For a given level of the treatments, Cd accumulated the most in root and least in leaf tissues. For the same tissue, the higher the Cd concentration of treatments, the higher the concentration absorbed by the tissue. The highest Cd concentration (2991.17 mg/kg) was detected in roots, which were almost four folds than in leaves under the treatment of Cd50, 16.7-fold than in leaves (179.43 mg/kg) under treatment of Cd10. The differences in Cd concentration between different tissues under the same Cd treatment or in the same tissue under different treatments reached extremely significant levels.

### Characteristics of primary structure and cytology of roots

**Figure 5:**
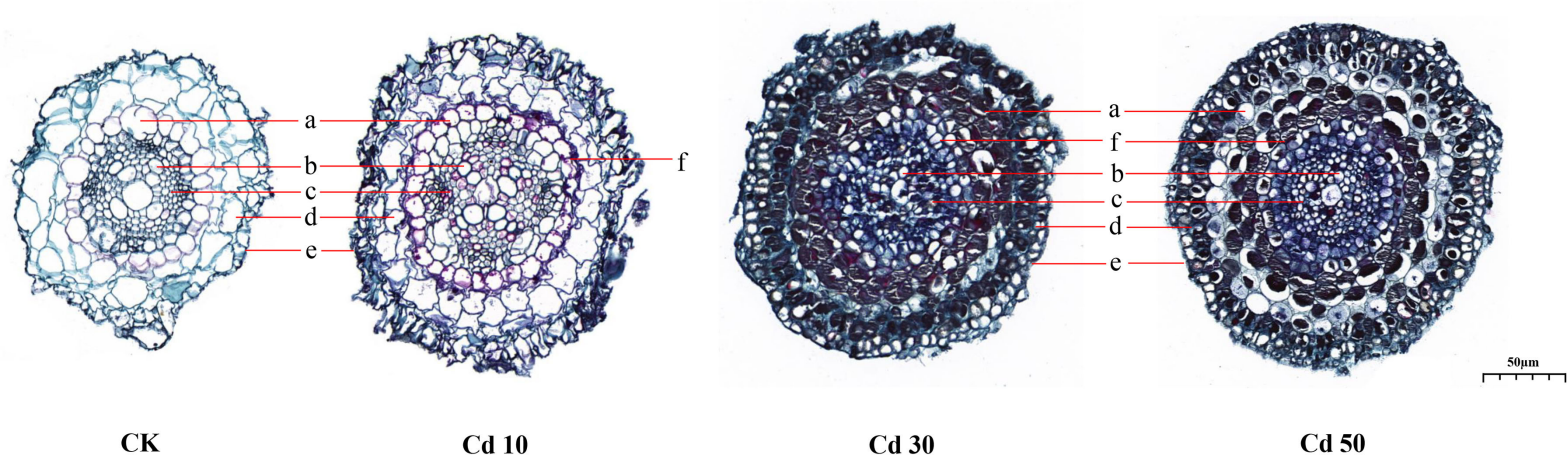
The primary structure of roots under different cadmium treatments. a, endodermis; b, primary xylem; c, primary phloem; d, cortex; e, epidermis; f, casparian strip. CK, Cd10, Cd30, Cd50 indicates the Cd concentration 0 mg/L, 10 mg/L, 30 mg/L, and 50 mg/L, respectively.

The significant differences in the primary structure of roots between CK and Cd treatments were identified (**Figure 5**). The transverse sections of roots from CK were smaller than those from Cd treatments. Compared with those from CK, the parenchymal cells in the cortex were smaller with increased numbers and closer arrangement under Cd treatments. Meanwhile, Cd treatments increased the endodermis thickness and reduced the sizes of primary xylems. Casparian strips were formed in Cd-treated roots.

**Figure 6:**
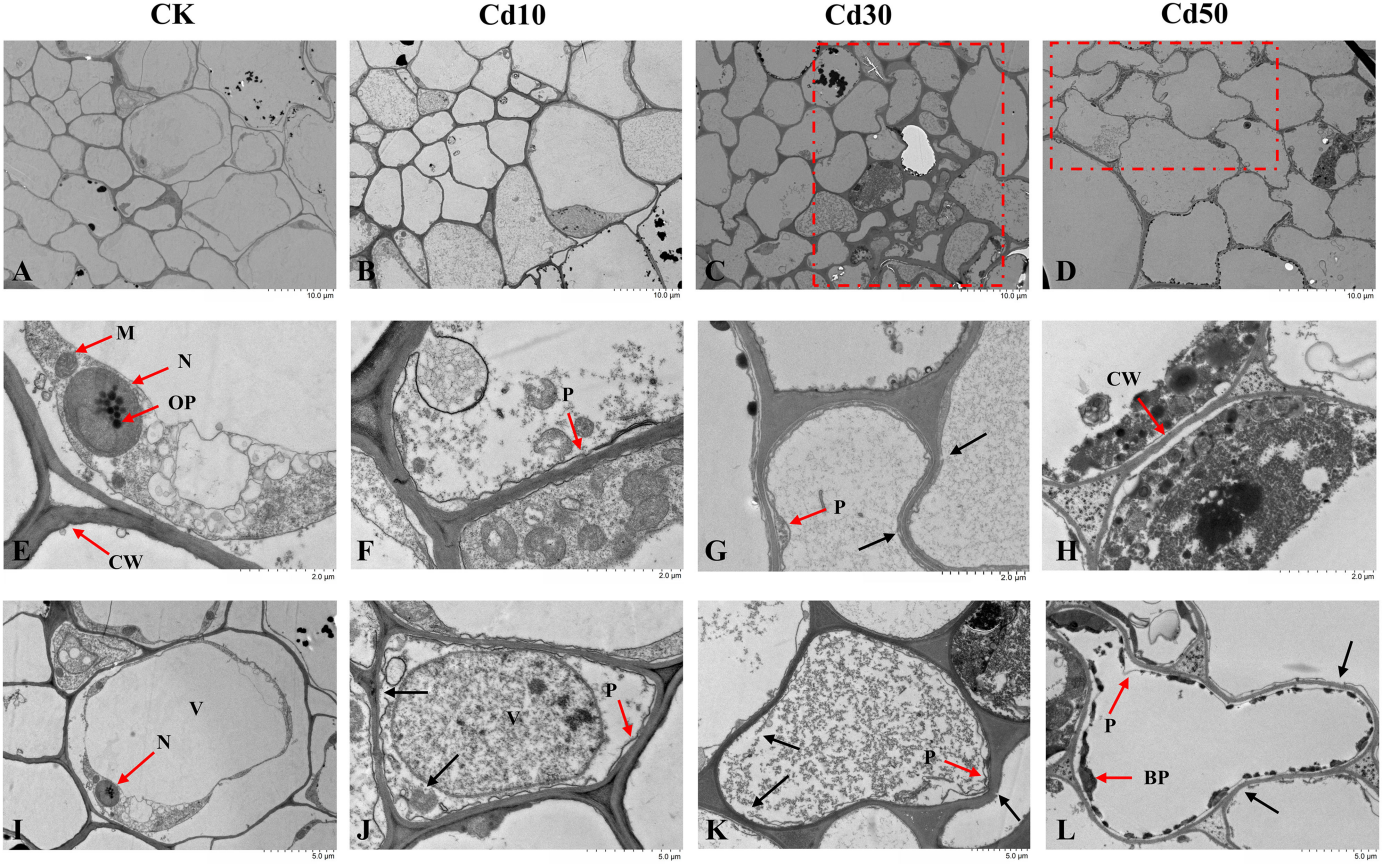
The ultrastructural examinations of root cells under different cadmium treatments. **(A, E, I)** Normal cell morphology, organelles, plasma membranes, and cell walls in controls. **(B–D)** Cell morphology under different Cd concentrations of treatments. **(F–H, J–L)** Damages induced by Cd in cell morphology, organelles, plasma membranes, and cell walls. CK, Cd10, Cd30, Cd50 indicates the Cd concentration 0 mg/L, 10 mg/L, 30 mg/L, and 50 mg/L, respectively. BP, black precipitates; CW, cell wall; M, mitochondria; N, nucleus; OP, osmiophilic particles; P, plasmolysis; V, vacuole. The red box shows the cell deformation. The red arrow points to the organelle or cellular content corresponding to the letters. The black arrow indicates the damaged parts.

The ultrastructural examinations showed that cells in roots from CK were well-shaped and complete with smooth plasma membranes, cell walls, and intact organelles (**Figures 6A, E, I** **)**. We observed the phenomenon of plasmolysis in root cells under Cd treatments (**Figures 6F, G, J–L**). The cells deformed under Cd30 and Cd50 (**Figures 6C, D, K, L** **)**. There’s no significant difference in cell walls between CK and Cd10, while the cell walls under Cd30 and Cd50 were slightly damaged (**Figures 6G, K, L** **)**, and under Cd50 were thinner than those under other treatments (**Figures 6D, H, L** **)**. The vacuoles enlarged, and many organelles were severely damaged and disappeared under Cd treatments (**Figures 6J–L**). The Cd-induced depositions were evenly distributed in the vacuoles under Cd10 and Cd30 (**Figures 6B, C, J, K** **)**. And some black precipitates gradually appeared on the inner sides of the vacuoles with the increase of Cd concentration (**Figures 6D, L** **)**.

### Transcriptome analysis of Tatary buckwheat under Cd treatments

To clarify gene expression of Tartary buckwheat under Cd stress, RNA sequencing was performed on roots treated with different Cd concentrations. A total of 78.61 Gb clean sequences were obtained from 12 samples, and each sample at least contained 6.10 Gb clean data. The score Q30 of the clean sequences for each sample was more than 94.17%. The clean data were mapped to the Tartary buckwheat reference genome, the mapping ratio varied from 90.30% to 93.67% for each sample, and the unique mapped reads accounted for 88.60-91.83% (**Supplementary Table 1**). A total of 31,674 transcripts were generated from all mapped reads. From the mapped results, we identified 1,956 new genes, 755 of them were functionally annotated. According to the threshold of average FPKM ≥ 1, 20,424, 20,700, 20,795, and 20,285 genes were expressed under CK, Cd10, Cd30, and Cd50, respectively. Of them, 377 genes were newly annotated, and 1,435 genes were highly expressed (average FPKM ≥ 100).

### Differentially expressed genes in response to Cd stresses

A total of 3,647 DEGs in response to Cd stresses were identified based on the criteria of FC ≥ 2 and FDR < 0.01 (**Table 1**). Compared with CK, 1,130 (726 up-regulated and 404 down-regulated), 1,581 (1,110 up-regulated and 471 down-regulated), and 3085 (1,798 up-regulated and 1,287 down-regulated) DEGs were identified from Cd10, Cd30, and Cd50, respectively. The results showed that the higher the Cd concentration, the more the DEGs. Moreover, 701 of these DEGs were detected in all of the Cd treatments. Of them, 495 were up-regulated and 206 down-regulated. There were 107 DEGs between Cd10 and Cd30, 1,108 between Cd10 and Cd50, and 920 between Cd30 and Cd50 (**Table 1**).

**Table 1:** The analysis of differentially expressed genes (DEGs).

Group	DEGs-total	DEGs-up	DEGs-down
CK vs Cd10	1130	726	404
CK vs Cd30	1581	1110	471
CK vs Cd50	3085	1798	1287
Cd10 vs Cd30	107	65	42
Cd10 vs Cd50	1108	594	514
Cd30 vs Cd50	920	327	593
CK vs Cd10&Cd30&Cd50	701	495	206

CK, Cd10, Cd30, and Cd50 indicates the Cd concentration 0 mg/L, 10 mg/L, 30 mg/L, and 50 mg/L, respectively. CK vs Cd10&Cd30&Cd50 indicates the DEGs existed in Cd10, Cd30, and Cd50 at once. Up and down indicate the up-regulated and down-regulated.

GO classification was carried out for the 3467 DEGs and all of the expressed genes. The DEGs could be categorized into 37 GO terms (**Supplementary Figure 1**). Compared with all expressed genes, a higher percentage of DEGs was involved in “single-organism process”, “localization”, “response to stimulus”, “multi-organism process”, and “immune system process” in the biological process category. A higher percentage of DEGs was expressed in “membrane”, “membrane part”, “extracellular region”, “cell junction”, “symplast”, “extracellular region part” in the cellular component category. A higher percentage of DEGs was enriched in “catalytic activity”, “transporter activity”, “antioxidant activity”, and “nutrient reservoir activity” in the molecular function category.

**Figure 7:**
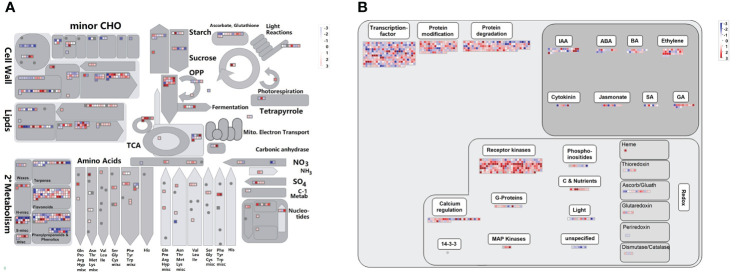
Overviews of cellular metabolism **(A)** and regulation **(B)** for DEGs. DEGs indicates differentially expressed genes. The individual small square indicates one DEG. The scale from -3 to +3 indicates the average FPKM normalized log2 transformed counts of DEGs.

To understand the gene functional characteristics of DEGs, we analyzed the overview of the metabolic and regulatory pathways. There were 546 DEGs mapped to the metabolism overview and 1017 DEGs mapped to the regulation overview (**Figure 7**). In metabolism overview (**Figure 7A**), most DEGs were involved in cell wall metabolism, lipids metabolism, secondary metabolism, and the oxidative pentose phosphate (OPP) pathway. In the secondary metabolism pathway, there were 65 DEGs related to flavonoids metabolism, and 73 were associated with phenylpropanoids & phenolic metabolism. In regulation overview (**Figure 7B**), a great number of DEGs were associated with transcription factor (**Supplementary Figure 2**), protein modification and degradation, hormone pathway, and signaling pathway. In the signaling pathway, 207 DEGs were receptor kinases, 36 DEGs were involved in calcium regulation.

### Core genes for Cd stress based on the gene co-expression network analysis

**Figure 8:**
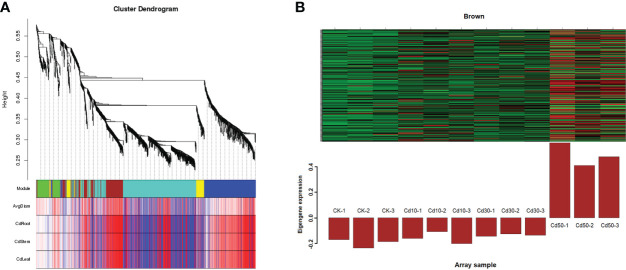
Weighted gene co-expression network analysis between traits and modules. **(A)** Heat map of correlations between modules and traits. In the traits part, the redder the color, the more positive the correlation. A strong correlation existed between genes in the brown module and the traits like ARD, and Cd concentration in different tissues. CK, Cd10, Cd30, Cd50 indicates the Cd concentration 0 mg/L, 10 mg/L, 30 mg/L, and 50 mg/L, respectively. AvgDiam, Average diameter; CdRoot, CdStem, and CdLeaf indicate the Cd concentration in roots, stems, and leaves, respectively. **(B)** The specific high expression genes under Cd50 treatments in the brown module.

**Figure 9:**
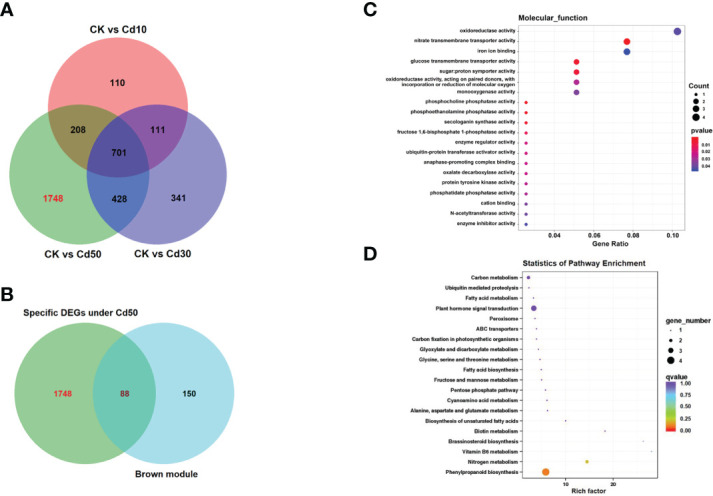
The Venn diagram, Molecular function, and KEGG (Kyoto Encyclopedia of Genes and Genomes) enrichment analyses for the hub genes induced by Cd50. **(A)** Venn diagram for the specifically expressed genes under Cd50. **(B)** The 88 genes were not only specifically expressed under Cd50 but also existed in the brown module. **(C, D)** Molecular function and KEGG analyses for 88 genes. CK, Cd10, Cd30, Cd50 indicates the Cd concentration 0 mg/L, 10 mg/L, 30 mg/L, and 50 mg/L, respectively. DEGs indicates differentially expressed genes.

After being filtered by the WGCNA package, a total of 1069 genes were used to generate a heat map of the module-sample matrix (**Figure 8A**). The results showed the brown module had higher positive correlations with root characteristic ARD and the Cd concentration in the three tissues assessed (**Figure 8A** and **Supplementary Figure 3**). The genes in the brown module were highly expressed under Cd50 treatment and not significantly different among CK, Cd10, and Cd30 (**Figure 8B**). The results suggested that genes specifically expressed under Cd50 treatments regulated ARD and Cd absorption. There were 159 genes in the brown module. Of them, 137 were selected to do further analysis based on the connectivity weight value over 0.7. Moreover, we identified 1748 DEGs that were specifically expressed under Cd50 treatments (**Figure 9A**). Of them, 88 were included in the brown module as well (**Figure 9B**). They were important genes specifically induced by Cd50. The molecular function analysis for the 88 genes showed that they were primarily involved in oxidoreductase activity, nitrate transmembrane transporter activity, and iron ion binding (**Figure 9C**). Furthermore, the KEGG pathway enrichment pathways were mostly phenylpropanoid biosynthesis and plant hormone signal transduction (**Figure 9D**).

**Figure 10:**
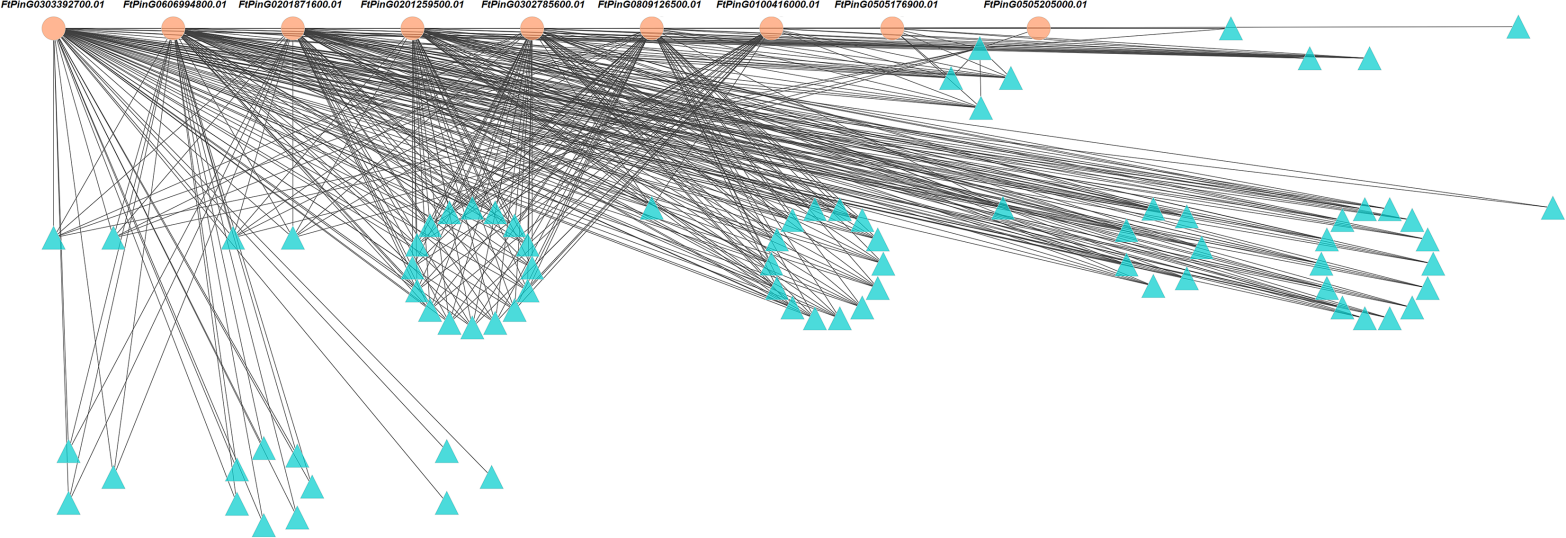
The interaction networks among 88 genes induced by Cd50 based on the gene expression profile. The genes with orange background were 9 core genes expressed under Cd50. Cd50 indicates the Cd concentration of 50 mg/L.

The interaction networks analysis showed 448 edges among the 88 nodes (**Figure 10**). We identified 9 core genes playing critical roles in the interaction networks based on the total weight values of connectivity (**Table 2**). They interacted with each other. Four of them were involved in metal ion binding. They were *FtPinG0201871600.01*, *FtPinG0505176900.01*, *FtPinG0505205000.01*, and *FtPinG0809126500.01*. *FtPinG0100416000.01* was homologous to *GMGT1* (galactomannan galactosyltransferase 1) in *Cyamopsis tetragonoloba*, which is involved in transferase activity in cell wall organization (Edwards et al., 2004). The function of *FtPinG0201259500.01* is unknown. *FtPinG0302785600.01* and *FtPinG0606994800.01* were transcription factors NAC and AFT, respectively. *FtPinG0302785600.01* was homologous to *Arabidopsis* gene *SMB* (*SOMBRERO*) involved in plant-type secondary cell wall biogenesis, positive regulation of cell fate commitment, regulation of transcription, and root cap development (Willemsen et al., 2008; Bennett et al., 2010). Transcription factor AFT (Gene ID: *FtPinG0606994800.01*) was involved in the positive regulation of transcription from RNA polymerase II promoter in response to iron ion starvation based on the GO annotation. *FtPinG0303392700.01* contained a calmodulin (CaM)-binding domain, which was related to the calcium (Ca) signal transduction pathway.

**Table 2:** The 9 core genes specifically induced by Cd concentration of 50 mg/L.

Gene ID	Log2(Fold change)	Annotation	Aligned species	Molecular function	Biological process
*FtPinG0100416000.01*	2.13	Galactomannan galactosyltransferase 1	*Morus notabilis*	Glycosyltransferase activity.	Cell wall organization.
*FtPinG0201259500.01*	1.81	Unknown	*Triticum aestivum*	–	–
*FtPinG0201871600.01*	2.01	Fructose-1,6-bisphosphate	*Beta vulgaris*	Fructose 1,6-bisphosphate 1-phosphatase activity. Metal ion binding.	Carbohydrate metabolic process.
*FtPinG0302785600.01*	1.95	Protein SOMBRERO	*Arabidopsis thaliana*	DNA binding. DNA-binding transcription factor activity.	Plant-type secondary cell wall biogenesis. Positive regulation of cell fate commitment. Regionalization. Regulation of transcription, DNA-templated. Root cap development.
*FtPinG0303392700.01*	1.82	Putative Calmodulin-binding domain	*Lupinus albus*	Calmodulin binding.	–
*FtPinG0505176900.01*	2.00	Peroxidase 57	*Arabidopsis thaliana*	Heme binding. Metal ion binding. Peroxidase activity.	Hydrogen peroxide catabolic process. Response to oxidative stress.
*FtPinG0505205000.01*	4.99	Cytochrome P450 76AD1	*Beta vulgaris*	Heme binding. Iron ion binding. Monooxygenase activity. Oxidoreductase activity, acting on paired donors, with incorporation or reduction of molecular oxygen.	–
*FtPinG0606994800.01*	2.86	Transcription factor AFT	*Macleaya cordata*	DNA-binding transcription factor activity, RNA polymerase II-specific. Methylmalonate-semialdehyde dehydrogenase (acylating) activity.	Positive regulation of transcription from RNA polymerase II promoter in response to iron ion starvation.
*FtPinG0809126500.01*	2.00	Endonuclease 1	*Arabidopsis thaliana*	Endonuclease activity. Metal ion binding. Nucleic acid binding. Double/single-stranded DNA exodeoxyribonuclease activity. T/G mismatch-specific endonuclease activity.	DNA catabolic process. Floral organ senescence. Leaf senescence. RNA phosphodiester bond hydrolysis, endonucleolytic.

### Validation of the core genes induced by Cd stress using qRT-PCR analysis

**Figure 11:**
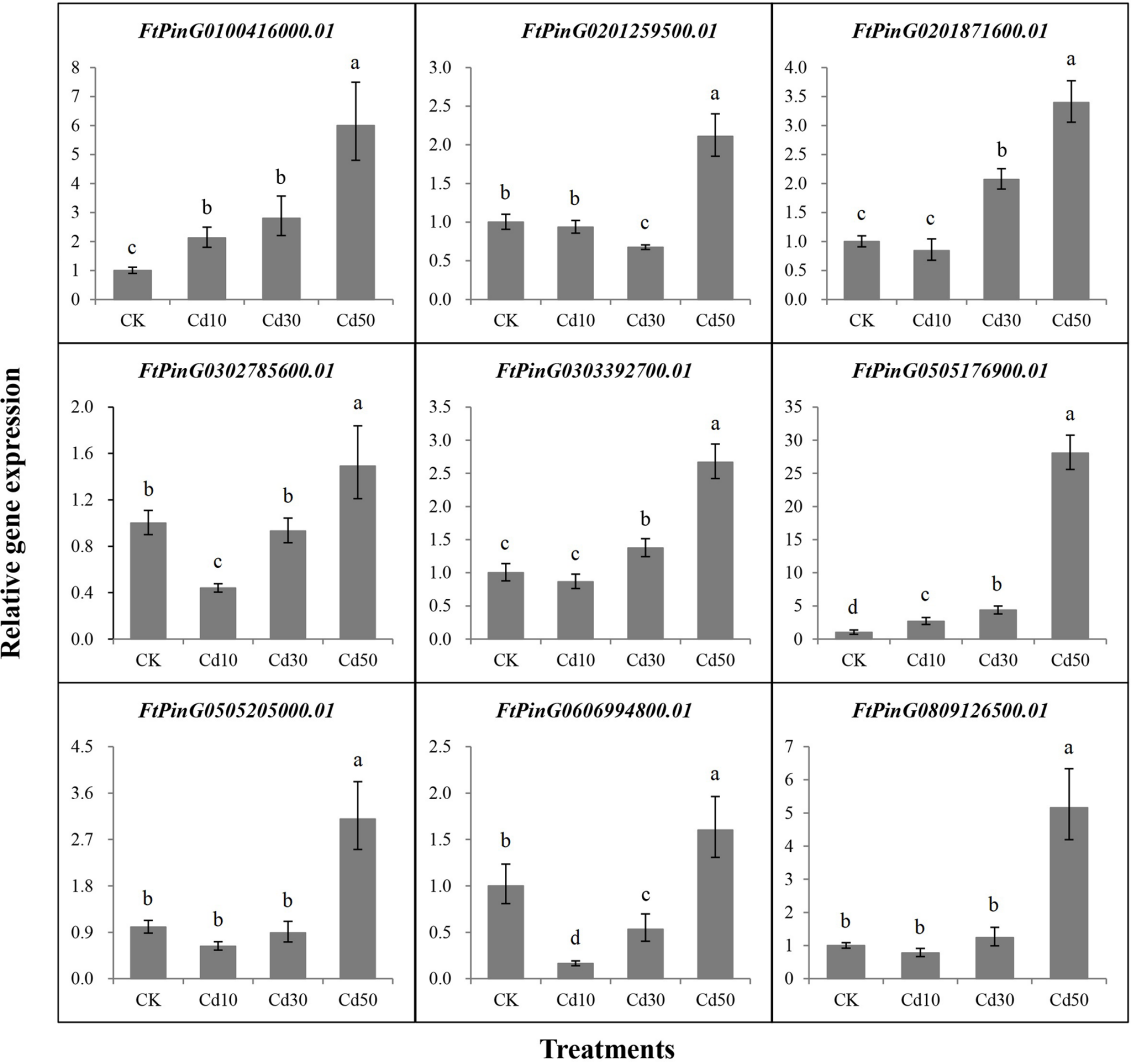
The relative gene expression for 9 core genes was highly induced by Cd stress of 50 mg/L. CK, Cd10, Cd30, and Cd50 indicates the Cd concentration 0 mg/L, 10 mg/L, 30 mg/L, and 50 mg/L, respectively. Different lowercase letters on the top of bars indicate significant differences between treatments according to Duncan *t*-test (*p*-value < 0.05).

Nine core genes played critical roles in the response to Cd stress based on the WGCNA and interaction networks analyses. To verify the expression levels of these genes, we designed primers (**Supplementary Table 2**) for qRT-PCR analysis based on their coding sequence (CDS). As displayed in **Figure 11**, a significant difference in expression for each gene was detected between Cd50 and the other treatments. The 9 core genes were expressed more under Cd50 than under any other treatment, which was consistent with the gene expression trend in the brown module as shown in **Figure 8B**. Meanwhile, the expression levels of *FtPinG0505205000.01* and *FtPinG0809126500.01* were the same as the RNA-seq results under different treatments. The expression for the rest seven genes slightly varied under Cd10 and Cd30. *FtPinG0100416000.01* showed increase expression under Cd10 and Cd30. *FtPinG0201259500.01* down-regulated under Cd30. *FtPinG0201871600.01* and *FtPinG0303392700.01* slightly upregulated under Cd30. The expression of *FtPinG0302785600.01* decreased under Cd10. With the increase of the Cd concentration, the expression of *FtPinG0505176900.01* enhanced. And its expression under Cd50 treatment is 28 times that of CK. *FtPinG0606994800.01* down-regulated both under Cd10 and Cd30.

## Discussion

### Comprehensive responses to Cd stress in Tartary buckwheat

Cd stress inhibited Tartary buckwheat growth. Although high concentrations of Cd accumulated (2991.17 mg/kg) in the roots, the plants showed no obvious disease spots even under the high concentration of Cd treatment (50 mg/L) for 7 days (**Figure 1**). The results seem to indicate that Tartary buckwheat cv XQ1 is highly tolerant to Cd, with the increase in root diameter as a possible contributing factor. Potocka and Szymanowska-Pułka (2018) reported that plants could increase root diameter to adapt to unfavorable environments. Many reports found that Cd stress can elevate the root diameter and accelerate the root maturation (Scebba et al., 2006; Lux et al., 2011; Wang et al., 2016; Qin et al., 2018). Here, we observed significant differences in root morphological characteristics and transverse sections between CK and Cd treatments. Compared with CK, the root diameter was larger because of more cell layers of the endodermis and the larger size of the pericycle (**Figure 5**). Maksimović et al. (2007) reported the bigger size of parenchyma cells and cortical tissues caused an increase in root diameter, thus improving plant resistance to water and solute flow under Cd stress. Bochicchio et al. (2015) believed that the increase in root diameter could be a compensatory growth under metal stress, and it’s a barrier to protect the root from the metals. Liu et al. (2020) found that rice plants had multiple strategies like altering root system architecture (including increasing root diameter) to enhance their tolerance to Cd-induced oxidative stress. Therefore, we believe the increase in root diameter may be a defense response to Cd toxicity in Tartary buckwheat. In addition, with the Cd treatments, the parenchyma cells in the cortex lined up tighter, and the Casparian strip was formed. We thought the relative less Cd transferred to the stems and leaves, which may be limited by the Casparian strip formed in the roots (**Figure 5**). As reported by Lux et al. (2011), the physical barriers like Casparian strip and lignification can limit Cd translocation to the shoots. Santoro et al. (2022) believed that the up-regulated gene involved in cell wall remodeling and lignification had the function of avoiding cadmium uptake. It’s probably another way for Tartary buckwheat to respond to Cd stress (El Rasafi et al., 2020).

Cd stress activated the antioxidant system as significant changes in antioxidant activities in different tissues under different concentrations of Cd treatments. The enzymatic and nonenzymatic antioxidants belong to the important defensive system that can minimize metal-induced oxidative damage in plants (Rizwan et al., 2017; Hussain et al., 2019; Shanmugaraj et al., 2019; Abedi and Mojiri, 2020; El Rasafi et al., 2020; Angulo-Bejarano et al., 2021). Besides, with the increase of Cd concentration in treatments, MDA content decreased, which indicated the oxidative damage caused by Cd to plants was reduced (El Rasafi et al., 2020). Rizwan et al. (2017) believed pakchoi can enhance the Cd tolerance by decreasing the production of MDA. Therefore, we thought Tartary buckwheat has the potential tolerance to Cd stress. As found in our study, there were no chlorosis and necrosis on plants even under a high concentration of Cd treatment for 7 days. Meanwhile, GO classification analysis showed a significantly higher percentage of DEGs involved in antioxidant activity and immune system process. We considered that the antioxidant system of XQ1 played a role in Cd detoxification.

Berni et al. (2019) believed that secondary metabolites like phenolic acids, flavonoids, and anthocyanins, together with the enzymatic antioxidant system, contributed to the counterbalance of ROS. Many transcription factors such as MYB, AP2/EREBP, WRKY, NAC, and bZIP played key roles in plant tolerance and mitigation of Cd stress (El Rasafi et al., 2020; Angulo-Bejarano et al., 2021). The receptor kinases were the important proteins that bound and transmitted signals, which can trigger the immune responses in plants (Zhou et al., 2019). In *Poa Pratensis*, many receptor kinases were highly expressed under Cd stress (Xian et al., 2020). All of these genes mentioned above were highly expressed in our study (**Figure 7**). The overviews of cellular metabolism and regulation pathways revealed most DEGs were involved in flavonoid metabolism, transcription factors, and receptor kinases, suggesting their potential roles in the Cd defense responses. To summarize, our results, for the first time, comprehensively analyzed the responses to Cd stress at phenotypic, physiological, cytological, and molecular levels in Tartary buckwheat.

### The function of 9 core genes in response to Cd stress

The cell wall is the first barrier for Cd to get into the cell and the major site to immobilize Cd by ion binding, which is an important strategy to detoxify Cd in plants (Shanmugaraj et al., 2019; Santoro et al., 2022). The *GMGT1* gene (Gene ID: *FtPinG0100416000.01*) is involved in galactomannan biosynthesis (Edwards et al., 2004). Galactomannans are the largest group of cell wall storage polysaccharides, which have the function of thickening, emulsifying, gelling, flocculating, and film-forming (Bento et al., 2013). In this study, the expression of *GMGT1* gene (Gene ID: *FtPinG0100416000.01*) may regulate the cell wall against the Cd stress (Bento et al., 2013). As shown in **Figure 6**, the cell wall under Cd50 was thinner than others.

The *FtPinG0201259500.01* is an uncharacterized protein, its function is unknown, but we can be sure it plays an important role in responding to Cd stress based on our studies. It’s a potential novel discover in the function of *FtPinG0201259500.01* to respond to Cd stress.

Cd stress can affect the expression of *FtPinG0201871600.01*, a photosynthetic enzyme in the cytoplasm, fructose-1,6-bisphosphatase. Song et al. (2019) reported that Cd stress can inhibit energy utilization and carbon sequestration by slowing down the activity of fructose-1,6-bisphosphatase. Here, we speculated the growth inhibition of Tartary buckwheat because the lower activity of fructose-1,6-bisphosphatase (Gene ID: *FtPinG0201871600.01*) caused by Cd stress can depress the photosynthesis and energy consumption.

The *SMB* gene (Gene ID: *FtPinG0302785600.01*) could repress stem cell-like divisions in the daughter cells of the root cap and increase the number of cell layers of columella and lateral root cap (Willemsen et al., 2008; Bennett et al., 2010). *SMB* located in the nucleus belongs to the NAC transcription factor family, which is a big family involved in abiotic stress, plant growth, and development (Yang et al., 2021). Cd stress inhibited plant growth. The roots were shorter and the lateral roots fewer. However, the root diameter increased significantly (**Figure 2**). Therefore, we thought high expression of *SMB* gene (Gene ID: *FtPinG0302785600.01*) may inhibit root elongation through repressing stem cell-like divisions in the root cap daughter cells. Meanwhile, it may increase the root diameter by increasing the cell layer number of the endodermis (**Figures 2**, **5**). The increased cell layer numbers help to prevent the invasion of Cd (Bochicchio et al., 2015; Liu et al., 2020). This phenomenon was also found in many other plants like wheat, soybean, *Arabidopsis*, *Miscanthus sinensis*, and *Iris lactea* (Scebba et al., 2006; Lux et al., 2011; Bochicchio et al., 2015; Wang et al., 2016; Qin et al., 2018; Liu et al., 2020).

Cd stress can activate signaling systems in Tartary buckwheat. The calcium ion (Ca^2+^) is a major signal molecular under stress conditions (Zhu, 2016; Wang et al., 2021). *FtPinG0303392700.01* contains a CaM-binding domain. It could combine with Ca^2+^ to form a Ca^2+^-CaM complex, which can transduce the Ca^2+^ signal by regulating the activities of several downstream target proteins (Wang et al., 2021). Several studies have shown that the Ca^2+^-CaM complex is involved in the responses to Cd stress (Rivetta et al., 1997; Perfus‐Barbeoch et al., 2002; White and Broadley, 2003; Zeng et al., 2015). Moreover, WGCNA identified that the 88 genes including core genes associated with Cd concentration, suggesting the expression of *FtPinG0303392700.01* is associated with the concentration of Cd accumulation in roots. This result was supported by Shukla et al. (2014) since the gene *OsACA6* having the CaM-binding domain can sequestrate more Cd^2+^ in roots to maintain cellular ion homeostasis and modulate the ROS-scavenging pathway.

*FtPinG0505176900.01* is orthologous to *Arabidopsis* gene *peroxidase 57*, which can sense the signaling of Ca^2+^ and respond to oxidative stress. It also resisted the Cd stress by removing H_2_O_2_ in peroxidase activity in rice (Shah et al., 2001). In this study, the change of the antioxidant activity (**Figure 3**) in Tartary buckwheat may indicate the *peroxidase 57* gene (Gene ID: *FtPinG0505176900.01*) regulated the response to Cd stress by removing H_2_O_2_.

*FtPinG0505205000.01* is homologous to *Beta vulgaris* gene *CYP76AD1* (*Cytochrome P450 76AD1*), which was located in the membrane and involved in the pathway betalain biosynthesis, iron ion binding, and oxidoreductase activity (Hatlestad et al., 2012). In *Hylocereus monacanthus*, the *CYP76AD1* was not only can present different colors but also play fundamental roles in plant responses and adaptation to biotic and abiotic stresses (Zhang et al., 2021b). Here, the *CYP76AD1* gene (Gene ID: *FtPinG0505205000.01*) may regulate the Tartary buckwheat stem turning red under Cd stress (**Figure 1**) and taking part in the responses to Cd.

The GO annotation showed transcription factor AFT (Gene ID: *FtPinG0606994800.01*) is involved in the positive regulation of transcription from RNA polymerase II promoter in response to iron (Fe) ion starvation. Besides, He et al. (2017) reported that Cd uptake could lead to competition for the absorption of mineral nutrients like Fe. Therefore, we predicted the Cd stress may trigger the expression of transcription factor AFT (Gene ID: *FtPinG0606994800.01*) to deal with iron ion starvation.

Long-term high concentration Cd stress could damage the DNA, growth, and development, resulting in senescence and plant death (El Rasafi et al., 2020). *FtPinG0809126500.01* is orthologous to *Arabidopsis* gene *ENDO1* (*Endonuclease 1*) which was involved in the metal ion binding, nucleic acid binding, T/G mismatch-specific endonuclease activity, programmed cell death (PCD), and senescence in plants (Pérez-Amador et al., 2000; Farage-Barhom et al., 2008).

### Possible pathway underlying response of Tartary buckwheat to Cd stress

**Figure 12:**
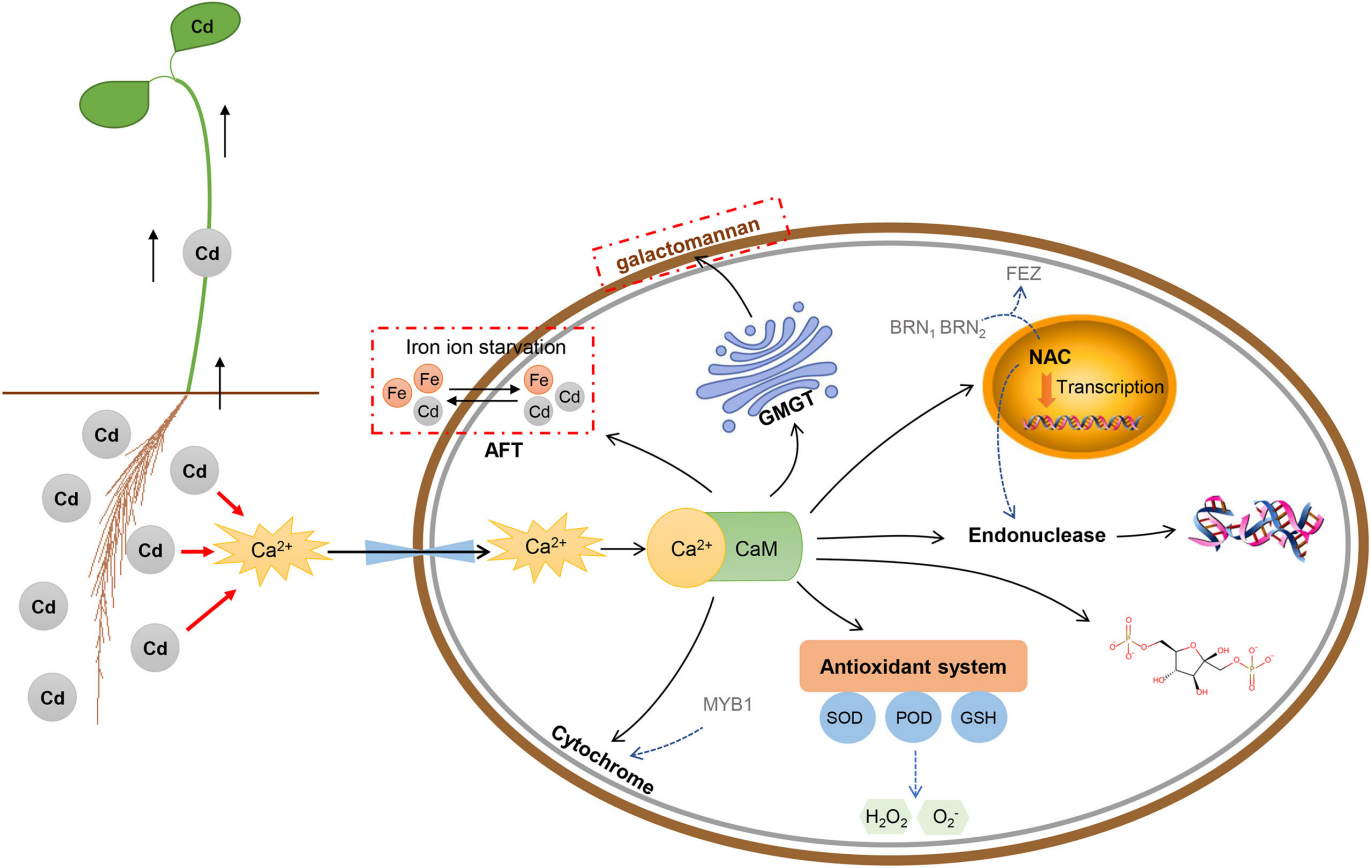
The schematic representation of the physiological and molecular processes induced by Cd stress in Tartary buckwheat roots cells. The CaM-binding domain can transduce the Ca^2+^ signal by combining with Ca^2+^ to form a Ca^2+^-CaM complex, then trigger a series of reactions downstream like antioxidant activity, metal ion binding, plant-type secondary cell wall biogenesis, regulation of transcription, and nucleic acid binding.

In this study, we analyzed the effects of Cd stress on phenotypic, cytological, physiological, and transcriptomic characteristics in Tartary buckwheat. The interaction networks of 9 core genes revealed that Cd stress can activate a series of responses in this species (**Figures 10**, **12**). Roots were the tissues in direct contact with Cd. Cd stress can activate the signaling system of Ca^2+^ to form the Ca^2+^-CaM complex with *FtPinG0303392700.01*. The Ca^2+^-CaM complex can trigger the downstream target proteins to respond to Cd stress (Shukla et al., 2014; Wang et al., 2021). The *Peroxidase 57* gene (Gene ID: *FtPinG0505176900.01*) took part in the hydrogen peroxide catabolic process to relieve the damage caused by Cd-induced H_2_O_2_. The *GMGT1* gene (Gene ID: *FtPinG0100416000.01*) could strengthen the cell wall to block the Cd invasion by regulating the galactomannan biosynthesis. The *SMB* gene (Gene ID: *FtPinG0302785600.01*) elevated the root diameter by increasing the number of cell layers and the size of the pericycle, which was a defense response to Cd stress by strengthening the root system. The *CYP76AD1* gene (Gene ID: *FtPinG0505205000.01*) is a cytochrome gene regulating the colors and it’s highly expressed under Cd50. The stems turning red under Cd stress may be regulated by this gene, which could respond to Cd stress by acting as oxidoreductase as well. In addition to the typical defensive responses to Cd stress above, Cd stress also affected the ion balance, and plant growth and development. The Cd uptake could result in competition for the Fe absorption (He et al., 2017). The transcription factor AFT (Gene ID: *FtPinG0606994800.01*) was involved in the regulation of iron ion starvation. Cd stress limited energy utilization and carbon sequestration by slowing down the activity of fructose-1,6-bisphosphatase (Gene ID: *FtPinG0201871600.01*), thus depressing the photosynthesis and energy consumption, inhibiting Tartary buckwheat growth (Song et al., 2019). Finally, the long-term high concentration of Cd stress can cause DNA damage like T/G specific mismatch regulated by *ENDO1* gene (Gene ID: *FtPinG0809126500.01*), leading to PCD and senescence in plants (Pérez-Amador et al., 2000; Farage-Barhom et al., 2008).

### Potential roles of Tartary buckwheat in Crop production and Cd phytoremediation

Tartary buckwheat can be developed into various commodities well received by consumers. The demanded amount of Tartary buckwheat is large. However, Cd contamination has occurred in many buckwheat growing areas in recent years (Huang et al., 2019). It not only can decrease the yield but also damage the quality. Growing resistance varieties is an eco-friendly, efficient, and sustainable approach against Cd stress. Understanding the Cd toxicity to plants was the first step to preventing and controlling Cd pollution. Xiqiao No. 1 used in this study is a widely cultivated variety in China, which has the advantage of early maturity and high yield. The Cd concentrations used in studying various species varied from 2 to 500 uM or from 3.3 to 100 mg/kg, the Cd concentration accumulated in roots ranged from 1.5 to 5000 mg/kg. Cd concentrations of roots obtained from most of the studies varied from 100 to 1000 mg/kg and only two of the species (Medicago sativa, and Viola) over 3000 mg/kg (Fan et al., 2021). Our study showed the roots of Tartary buckwheat contained a high concentration (2991.17 mg/kg) of Cd and that no chlorosis and necrosis showed in plants, indicating that this species has a high level of tolerance to Cd. It’s elite germplasm for further study of the Cd tolerance mechanism.

Phytoremediation is a way to use plants and associated soil microbes to reduce the concentrations and thus toxic effects of contaminants in the environment (Ali et al., 2013). This approach is environment-friendly, economically feasible, and highly efficient. Suitable plants used for phytoremediation should be characterized by a high accumulation of heavy metals and tolerance to them (Ali et al., 2013; Zhang et al., 2021a). In this study, high levels of Cd accumulation in roots and high tolerance to Cd may suggest the potential of Tartary buckwheat in the phytoremediation of Cd. Meanwhile, this species is a short-season crop with wide adaptability that can withstand cold and barren environments (Zhou et al., 2018). Tartary buckwheat, used as phytoremediation, can be planted in a wide range of environments and even grown twice a year in some warm (temperature above zero) places. Further, fertilizers and pesticides are usually not required for growing Tartary buckwheat. The comprehensive study of response to Cd stress in this study suggested Tartary buckwheat is a potential species in Cd phytoremediation.

## Conclusion

This study comprehensively identified the responses to Cd stress in Tartary buckwheat for the first time. A high concentration of Cd accumulated in roots, but there was no chlorosis and necrosis in plants, indicating a high tolerance to Cd stress. Root diameter increased as a result of the increase of the cell layer number and the pericycle size under Cd stress. The antioxidant systems were triggered as well. Nine core genes took part in the defense activities like metal ion binding, Ca signal transduction, cell wall organization, plant-type secondary cell wall biogenesis, hydrogen peroxide catabolic process, antioxidant activities, carbohydrate metabolic process, DNA catabolic process, and plant senescence. It provided the references for a deep understanding of the responses to Cd toxicity and genetic improvement in Tartary buckwheat. These results also provide a critical reference for the functional characterization of genes for Cd tolerance.

## Data availability statement

The datasets presented in this study can be found in online repositories. The names of the repository/repositories and accession number(s) can be found below: https://www.ncbi.nlm.nih.gov/, PRJNA755504.

## Author contributions

XY conceived and performed the experiments, analyzed the data, and wrote the manuscript. QL and CL analyzed the data. DX conceived the experiment. All the authors reviewed and edited the manuscript. All authors contributed to the article and approved the submitted version.

## Funding

This work was supported by National Key Research and Development Program of China (2019YFD1001300,2019YFD1001302), Sichuan Science and Technology Program (2022NSFSC1725), and the earmarked found for China Agriculture Research System (CARS-07-B-1).

## Conflict of interest

The authors declare that the research was conducted in the absence of any commercial or financial relationships that could be construed as a potential conflict of interest.

## Publisher’s note

All claims expressed in this article are solely those of the authors and do not necessarily represent those of their affiliated organizations, or those of the publisher, the editors and the reviewers. Any product that may be evaluated in this article, or claim that may be made by its manufacturer, is not guaranteed or endorsed by the publisher.

## Supplementary material

Supplementary Figure 1 Gene ontology classification of differentially expressed genes. *, the higher percentage of genes.

Supplementary Figure 2 Histogram of the number of transcription factors for differentially expressed genes.

Supplementary Figure 3 Heatmap of the Module-Trait correlation. AvgDiam, Average diameter; CdRoot, CdStem, and CdLeaf indicate the Cd concentration in roots, stems, and leaves, respectively.
