# Supplementary figures and images for: Transcriptomic, cytological, and physiological analyses reveal the potential regulatory mechanism in Tartary buckwheat under cadmium stress
Source: Front Plant Sci. 2022 Oct 12;13:1004802. doi: 10.3389/fpls.2022.1004802 (PMC9597304; doi:10.3389/fpls.2022.1004802)

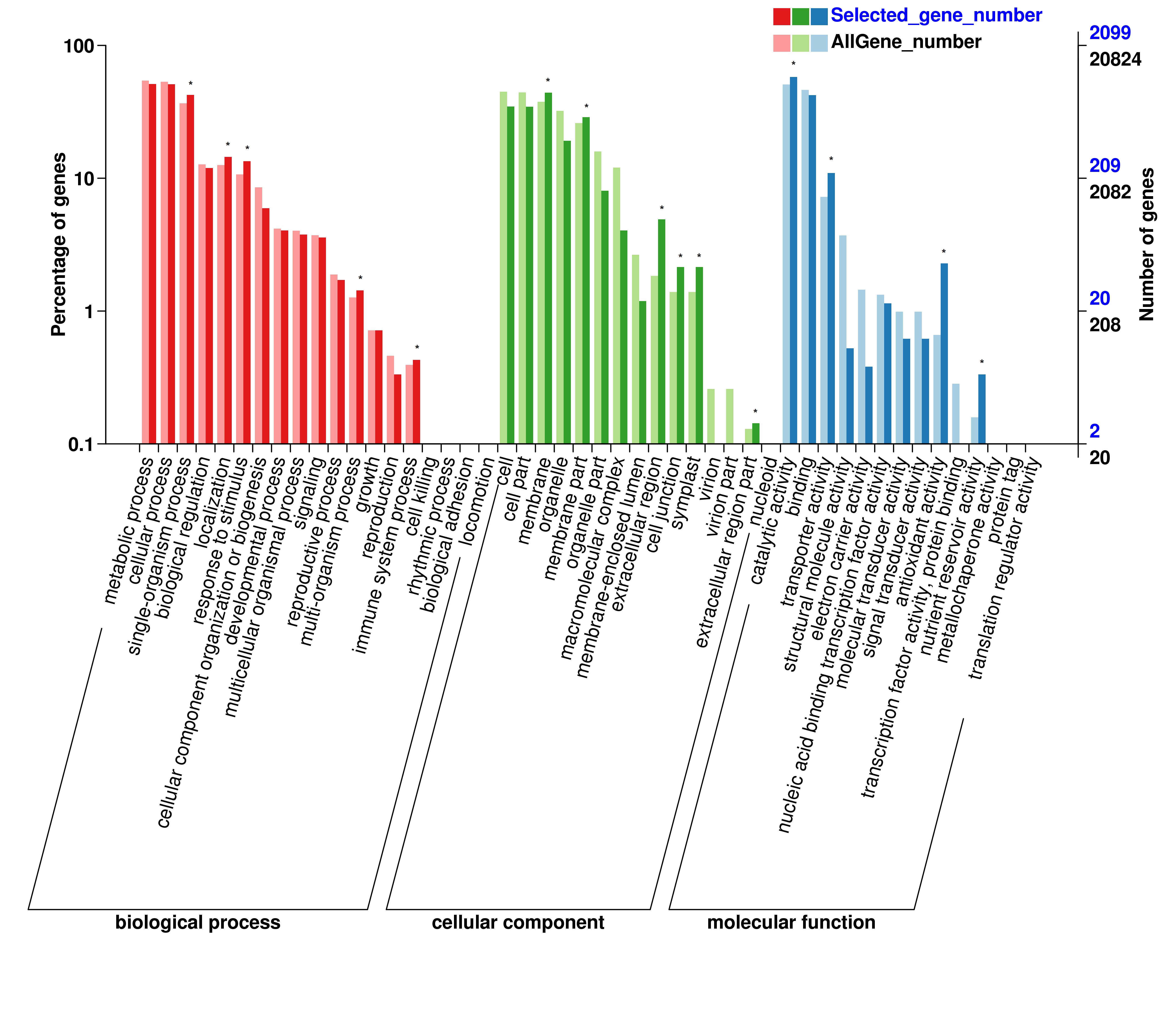

Supplement: Supplementary Figure 1 — Gene ontology classification of differentially expressed genes. *, the higher percentage of genes. [file Image_1.jpeg]

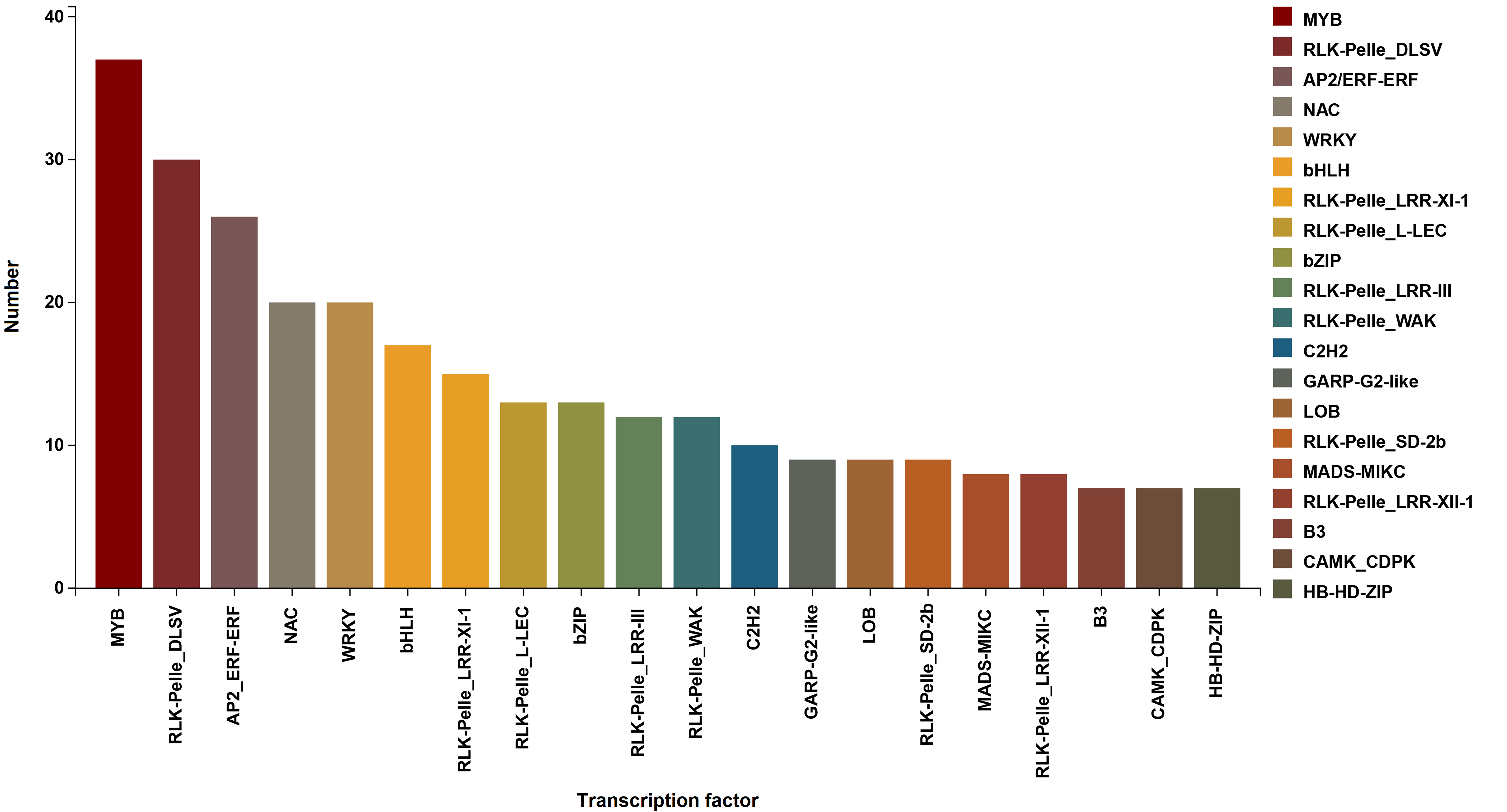

Supplement: Supplementary Figure 2 — Histogram of the number of transcription factors for differentially expressed genes. [file Image_2.jpeg]

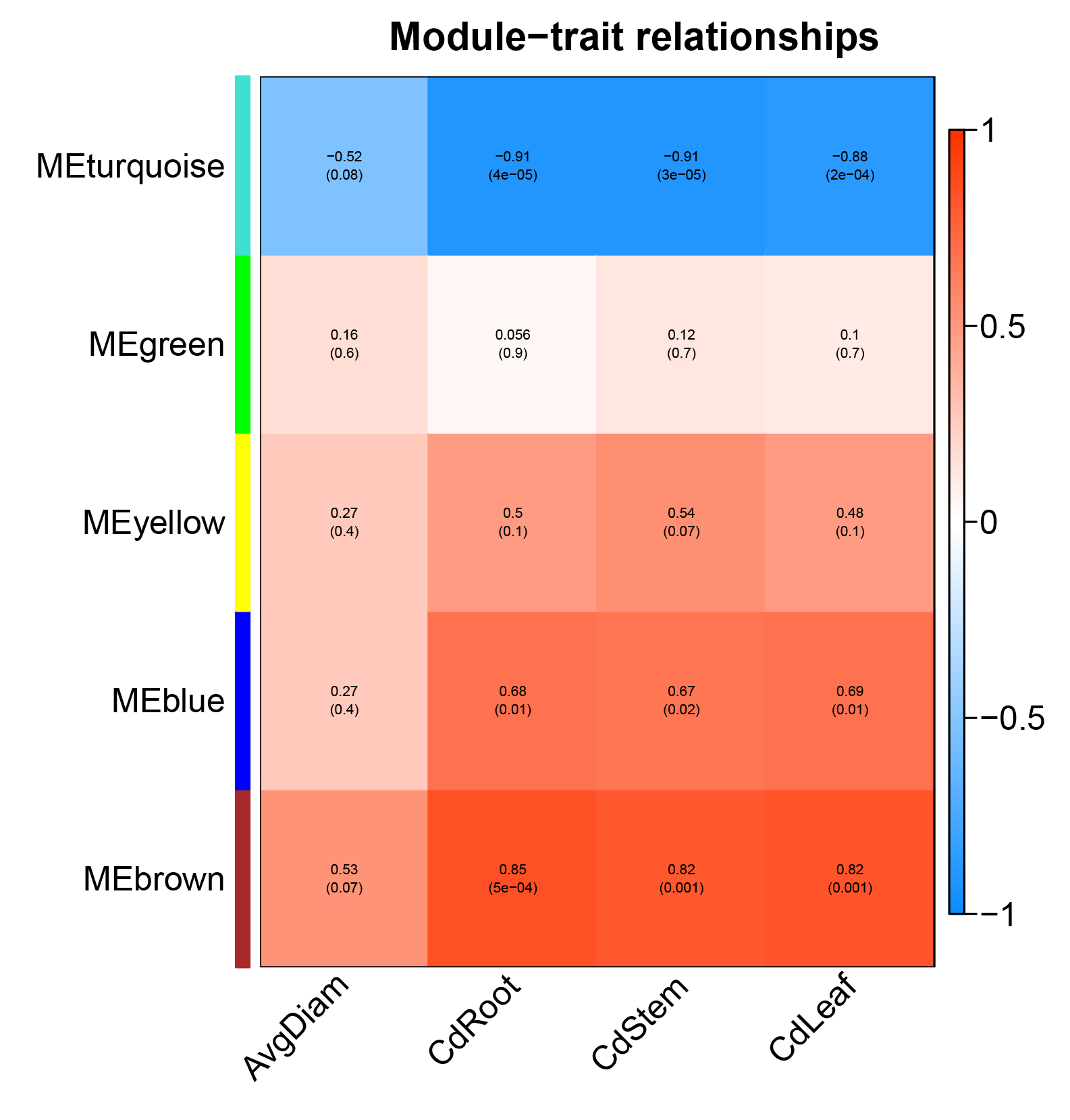

Supplement: Supplementary Figure 3 — Heatmap of the Module-Trait correlation. AvgDiam, Average diameter; CdRoot, CdStem, and CdLeaf indicate the Cd concentration in roots, stems, and leaves, respectively. [file Image_3.jpeg]
